# Detection of *EGFR* and *KRAS* gene mutations using suspension liquid-based cytology specimens in metastatic lung adenocarcinoma

**DOI:** 10.18632/oncotarget.22530

**Published:** 2017-11-20

**Authors:** Huan Zhao, Tian Qiu, Huiqin Guo, Jianming Ying, Junling Li, Zhihui Zhang

**Affiliations:** ^1^ Department of Pathology, National Cancer Center, Cancer Hospital, Chinese Academy of Medical Sciences and Peking Union Medical College, Beijing, China; ^2^ Department of Oncology, National Cancer Center, Cancer Hospital, Chinese Academy of Medical Sciences and Peking Union Medical College, Beijing, China

**Keywords:** EGFR, KRAS, lung cancer, adenocarcinoma, fine-needle aspiration

## Abstract

**Background:**

The detection of *EGFR* and *KRAS* mutations of metastatic lung adenocarcinoma using liquid-based cytology suspension routine specimens from fine-needle aspiration remains controversial.

**Results:**

The DNA of all specimens was extracted and real time PCR was performed successfully. The rate of *EGFR* and *KARS* mutations was 37.7% (58/154) and 5.8% (9/154), respectively. *EGFR* mutation rate was significantly higher in females than that in males (47.8% vs. 29.4%, *P* = 0.019). There were no significant differences among different age groups or different tumor sites. These results of *EGFR* and *KRAS* mutations using LBC specimens were consistant with the tissue samples. In 30 patients treated with tyrosine kinase inhibitors, complete response, partial response, stable disease and progress disease was observed in 2, 10, 13 and 5 patients, respectively.

**Conclusions:**

Liquid-based cytology specimen is reliable and can be an alternative source for the detection of *EGFR* and *KRAS* mutations.

**Methods:**

154 fine-needle aspiration cytologic samples were obtained from patients with metastatic lung adenocarcinoma. The specimens included 21 cases of mediastinal lymph node 123 cases of neck nodules and 10 cases of subcutaneous nodules. After the diagnosis and count of tumor cells performed by cytopathologists, liquid-based cytology specimens with sufficient tumor cells were used for *EGFR* and *KRAS* testing using real-time PCR.

## INTRODUCTION

Non-small cell lung cancer (NSCLC) with epithelial growth factor receptor (*EGFR*) mutation demonstrates increased sensitivity to the anti-*EGFR* tyrosine kinase inhibitors (TKIs) [1]. *EGFR* TKIs are now first-line therapy in patients who have advanced-stage lung adenocarcinoma with *EGFR* mutation [2]. In routine clinical practice, the tissue sample is usually required for *EGFR* and *KRAS* detection. However, lung cancer is sometimes diagnosed at an advanced stage, when these patients are not surgical candidates and tissue biopsies are not always available. The cytological materials may be the only source for the analysis of *EGFR* and *KRAS* mutations. Fine needle aspiration (FNA) is less harmful, more easily performed and more economicalthan other diagnostic technologies, which is useful in the diagnosis of metastatic lung cancer in lymph node. Some studies indicated that cytological specimens were suitable for *EGFR* and *KRAS* testing in patients with NSCLC using different specimens, such as cell block, liquid-based cytology (LBC) or conventional smears and fresh tumor cells [3–9]. The international guideline for the testing lung cancer biomarker also recommends cytological samples suitable for *EGFR* test [10, 11]. Hitherto, there were few studies exploring the *EGFR* detection in the metastatic foci of lung cancer using LBC samples of FNA, which is commonly encountered in practice. Therefore, it is necessary to establish easy and adequate method to get enough tumor cells for molecular detection.

Real-time polymerase chain reaction (PCR) is a high sensitivity method for the detection of gene mutation compared with direct sequencing. The *EGFR* and *KRAS* gene mutation can be detected in samples containing as few as 1% tumor cells [5, 6]. Despite of this, *EGFR* and *KRAS* testing in low-cellularity specimen is still being questioned. Therefore, in addition to the selection of sensitive detection methods, the amount of cell samples is also very important to gene detection. The cytological samples were usually fixed and prepared in different ways, such as direct smear, cell block and liquid based preparation. The methods achieving cytological specimens for *EGFR* detections were as follows: scraping tumor cells from smear slides by manual or laser microdissection [7, 8], cutting slides from cell block [3], or using fresh tumor cells from FNA [9]. These methods had both advantages and disadvantages. Gene detection can be performed successfully if there are enough tumor cells, but there is a certain cell waste using cell block and scrape methods if the tumor cell content is low.

LBC technology not only assists to make cytological diagnosis but also can preserve cells. LBC has potential advantages when the material is used for molecular detection. The homogenous cell distribution facilitates assessment of the percentage of malignant cells in the specimens [12]. Some studies proved thatLBC specimens of FNA can be used to perform some molecular tests [13–15]. Herein, we selected sensitive method (real-time PCR) to detect *EGFR* and *KRAS* gene mutations using the suspensions of LBC from FNA samples of metastatic lung adenocarcinoma. We wish to find an optional and reliable resource of cytological specimens for *EGFR* and *KRAS* gene detection. The aim of this study is to explore the feasibility and reliability of this approach.

## RESULTS

### Patients

A total of 154 cellular samples were obtained from our department. The molecular test was successfully performed in all specimens. Of these samples, 21 samples arised from mediastinal lymph node, 123 from neck nodules and 10 from subcutaneous nodules. There are 85 males and 69 females in our study. The age ranged from 28 to 87 and the average age was 55.4 years old.

### *EGFR* and *KRAS* mutations

**Figure 1:**
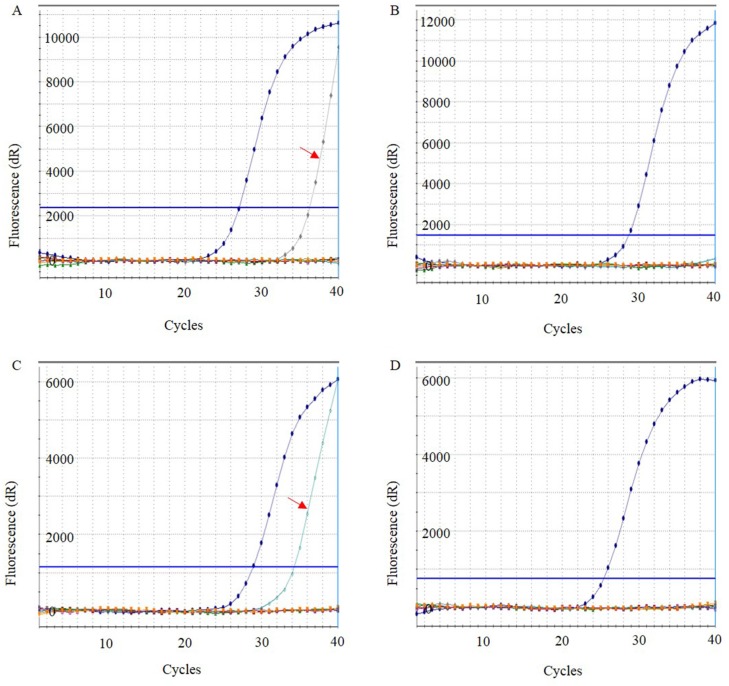
Detection of *EGFR* and *KRAS* mutations using suspension liquid-based cytology specimens from fine-needle aspiration in metastatic lung adenocarcinoma by real-time PCR Representative images of positive (**A**, **C**) and negative (**B**, **D**) for *EGFR* and *KRAS* mutations by real-time PCR. Positive curve were indicated by red arrows.

Table 1 shows the results of *EGFR* mutations in FNA samples. Of 154 adenocarcinoma, *EGFR* mutation rate is 37.7% (58/154). Most of the *EGFR* mutations are located at exons 19 and 21, which account for 44.8% and 41.4%, respectively. *KRAS* mutation rate is 5.8% (9/154) (Figure 1). 4 cases show concurrence of 2 mutation points in *EGFR* gene. No mutation was simultaneously found in *EGFR* and *KRAS*.

**Table 1:** *EGFR* and *KRAS* mutations of metastasis lung adenocarcinoma

*EGFR*	Exon	Mutations sites	*N*
	18	c.2155G>A/T, c.2156G>C (p.G719S/C/A)	3
	19	del2233-2247, 2235-2246, 2235-2249, 2235-2251>AG, 2235-2251>AATTC, 2235-2252, 2235-2252>AAT, 2235-2255>AAT, 2236-2250, 2237-2251, 2235-2248>AATTC, 2236-2248>CAAC, 2236-2253, 2237-2254, 2237-2255>T, 2237-2256>TT, 2237-2256>TC, 2238-2248>GC, 2238-2252, 2238-2252>GCA, 2238-2255, 2238-2256>GCAA, 2239-2247, 2239-2248>C, 2239-2251>C, 2239-2253, 2239-2256, 2239-2256>CAA, 2239-2258>CA, 2240-2251, 2240-2254, 2240-2257	27
	20	c.2369C>T (p.T790M)	2
	20	c.2303G>T (p.S768I), c.2307-2308insGCCAGCGTG, c.2309-2310AC>CCAGCGTGGAT, c.2310-2311insGGT, 2311-2312insGCGTGGACA, 2319-2320insCAC	1
	21	c.2573T>G, c.2573-2574TG>GT (p.L858R)	20
	21	c.2582T>A (p.L861Q)	5
*KRAS*	12 codon	c.34G>T (p.G12C)	4
	12 codon	c.34G>A (p.G12S)	0
	12 codon	c.34G>C (p.G12R)	0
	12 codon	c.35G>T (p.G12V)	1
	12 codon	c.35G>A (p.G12D)	4
	12 codon	c.35G>C (p.G12A)	0
	13 codon	c.38G>A (p.G13D)	0

Table 2 shows the correlation between *EGFR* gene mutations and clinic-pathologic parameters. The *EGFR* mutations rate was 47.8% (33/69) in females and significantly higher than that in males 29.4% (25/85) (*P* = 0.019, χ^2^ = 5.500). There were no significant differences among different age groups and different puncture sites. There were also no differences *EGFR* mutation rate in different age groups which were classified by 10 years old (Table 3), (*P* = 0.555).

**Table 2:** Patient characteristics and frequency of *EGFR* mutations of metastasis lung adenocarcinoma in FNA sample

Characteristic	All case	*EGFR* mutations (+)		*P* value
	*n* = 154	(*n* = 58)		
Age				
Range		31–75		
mean ± SD		56.28 ± 10.24	*t* = 0.723	0.209
Gender				
Female	69	33 (47.8%)	χ^2^ = 5.500	0.019^*^
Male	85	25 (29.4%)		
Site				
EBUS-FNA (LN)	21	7 (33.3%)	χ^2^ = 0.515	0.773
Neck nodule	123	48 (39.0%)		
Subcutaneous nodule	10	3 (30.0%)		

**Table 3:** Different age group and *EGFR* mutation rate

Age group	mutation rate (*n*/*N*)
≤40	23.1% (3/13)
41–50	35% (14/40)
51–60	45.2% (24/53)
61–70	32.1% (9/28)
≥71	40% (8/20)

*P* = 0.555

The *EGFR* and *KRAS* detection were performed in nineteen pairs of samples, which includ both LBC samples and FFPE samples. Of these 19 pairs of samples, ten pairs originated from the lymphonodes, each of them originated from the same site and had consistent results (kappa = 1.0). In another nine samples, the *EGFR* and *KRAS* mutation detection was performed in metastatic lesions of lymph node for liquid-based cytologic samples and in primary lesions for FFPE samples. They also showed the same results for the detection of gene mutation (kappa = 1.0).

### The curative effect of TKI

The clinical data suggested that 30 patients with *EGFR* gene mutations were treated with *EGFR* TKIs. Of these 30 patients, complete response (CR), partial response (PR), stable disease (SD) and progress disease (PD) was found in 2, 10, 13 and 5 patients, respectively (Table 4). Objective response rate (ORR) was 40% (12/30). The ORR in exon 19 deletion and exon 21 point mutations cases was 29.4% (5/17) and 50% (5/10), respectively. The ORR did not show significantly difference between patients with an exon 19 deletion and those with the exon 21 point mutations (*P* = 0.415, Fisher’s Exact Test).

**Table 4:** The relationship between *EGFR* gene mutations and curative effect in 30 patients

	Mutations	CR	PR	SD	Progress	Total
*EGFR*	19	1	4	9	3	17
	21	1	4	3	2	10
	18 and 20	0	1	0	0	1
	19 and 21	0	1	1	0	2
total		2	10	13	5	30

Abbreviations: CR: complete response, PR: partial response, SD: stable disease.

## DISCUSSION

LBC technology can blend cells proportionally and uniformly, thus it can make tumor cells evenly distributed on the slides. Cytologists can evaluate the number of tumor cells at the same time of diagnosis. The number of tumor cells in the suspensions can be evaluated indirectly by the pap staining slide. If the suspensions were enough, molecular test could be done. In order to find a reliable and convenient resource of cytological specimens for *EGFR* gene detection, we selected the suspensions of LBC from FNA samples that were diagnosed as lung adenocarcinoma. In this study, the concentration of extracted DNA was more than 300 ng/µl in each sample, which ensured enough DNA for gene detection. The influence of cytolyte solution and preservation solution on DNA extraction in LBC suspensions will be carefully studied and evaluated in the future studies [12].

Compared to cell block, this method was easily performed and wasted few tumor cells. Meanwhile, the cell morphology can be well reserved. The cytopathologists only need to evaluate whether the tumor cell amount was sufficient only, which make it more practical. The remaining work was done in the molecular pathology laboratory. They would not need to select tumor cells and scratch or cut off the cells from the slides. Meanwhile, the evaluation of tumor content also has variation among different observers. The aim of this study was to extract DNA from the suspensions of LBC and to detect gene mutation. Therefore, we must ensure enough tumor cells to avoid the false negative. In this study, we did not explore the minimum cells volume for *EGFR* mutations analysis. One study indicated that even 100–1,000 tumor cells from FNA samples provided reliable results of mutations analysis when sensitive real time PCR method was used [16]. Another study indicated that the cytological sample with low (10–20%) content of tumor cells was also a sufficient source for *EGFR* mutation testing in NSCLC patients [6]. However, we consider that the number of tumor cells must be estimated rather than the tumor cells proportion only for molecular test, especially for low-cellularity specimens.

In clinical practice, the minimally invasive samplings, including small biopsy and cytological specimens, sometimes get few tumor cells, which is not enough for gene detection. Mutations in low-cellularity specimens were considered as a true positive finding, but it might result in a false-negative interpretation. And thus, the low-cellularity specimens were not suitable for clinical test. The adequate number of tumor cells is essential to guarantee the accuracy of the detection result. For palpable nodules, free-handed FNA is easily performed and another pass FNA for *EGFR* detection is more acceptable for patients. In this study, 133 samples were obtained by free-handed FNA. Re-sampling was required in about 1/3 cases. We recommended that two or more pass FNA were feasible to ensure adequate tumor cells for molecular test for palpable mass. When the cancer locations were inaccessible by other modalities or due to the patient’s poor physical condition, EBUS-TBNA was a practical and feasible method to obtain abundant tumor cells samples for diagnosis and molecular analysis [17]. We selected 21 EBUS-TBNA samples with sufficient tumor cells for testing of gene mutation. The results demonstrated that *EGFR* mutations rate was 30% (7 cases) and had no difference with that in body surface tumors. So, if tumor cells were sufficient, EBUS-TBNA cytological material would be an alternative source for *EGFR* detection.

In Asian patients, the rate of *EGFR* mutations was higher, while *KRAS* mutations rate was lower in lung adenocarcinoma compared with that in Westerns [5, 18, 19]. It was reported that the *EGFR* mutation rate of Western/ADC versus Asian/ADC was 19.2% (940/4890) vs. 47.9% (1492/3117). However, the *KRAS* mutations rate was 26.1% (613/2352) vs. 11.2% (236/2114) [19]. Our study showed that the mutation rates of *EGFR* and *KRAS* in metastatic lung adenocarcinoma were 37.7% and 5.8%, respectively. A recent study indicated the rate of *EGFR* mutations was 39.6% among the 10,687 Asia patients [20]. Our result suggested that most of the *EGFR* mutations were located at exons 19 and 21 (accounting for 44.8% and 41.4%, respectively), which was similar toprevious studies [21–22]. Our result showed the mutation rate of *EGFR* in female patients was 47.8%, which was significantly higher than that in male patients (29.4%). It was similar to the finding from India in females and males (50.5% vs. 33.9%) [7]. In this study, the *EGFR* and *KRAS* mutations had consistent results in cytologic samples and histological samples in the same lymph node and also consistent in metastatic lesion of lymph node and in primary lung lesion. However, further study with larger samples is required to confirm this result. Also, some studies reported that the frequency of *EGFR* gene mutations was similar between cytological and histological samples [5, 18]. A multicenter retrospective study showed *EGFR* mutations were detected with similar frequency in primary and metastatic sites [6]. This finding may facilitate testing of patients with metastatic lung adenocarcinoma for *EGFR* mutations with minimally invasive techniques, and thus avoiding more invasive procedures.

In our study, 30 patients with *EGFR* gene mutations received TKI targeted therapy. Of these 30 patients, CR, PR, SD and PD were found in 2, 10, 13 and 5 patients, respectively. ORR was 40.0%. It was reported, however, that in a study enrolling 230 patients from 43 institutions in Japan, the ORR was 73.7% [23]. The ORR in our study was lower than that reported by other studies [23, 24]. It may be associated with the schedule of evaluation and different clinical stages. Stratification analysis was not done due to the limited cases. The response rate did not differ significantly between patients with an exon 19 deletion and those with the exon 21 point mutations, which is similar to that reported by Maemondo M [23]. Further study should be performed to analyze the correlation between the *EGFR* mutations by FNA samples and efficacy.

## CONCLUSIONS

LBC suspension of FNA sample is a reliable and alternative source for *EGFR* and *KRAS* mutations detection using validated, sensitive real-time PCR method. FNA performance is less harmful and can be repeated, which is suitable for clinical application.

## METHODS

### Patients

From February 2011 to December 2014, the cellular samples of 154 advanced lung adenocarcinoma patients with lymph node metastasis were obtained from our institution. The FNA were performed by cytopathologists for palpable nodes. The FNA of mediastinal lymph nodes were performed by endoscopic doctors under the endobronchial ultrasound guided transbronchial needle aspiration (EBUS-TBNA). The diagnosis was mainly based on cytomorphological features. The immunocytochemistry staining was performed in some cases. The diagnosis of 40.3% (62/154) cases was confirmed by histopathology. Other cases had typical cytomorphological characteristics or immunocytochemistry features of adenocarcinoma with clinical and image evidence of lung cancer. This study were reviewed and approved by the ethics committee of the Cancer Hospital, CAMS. All patients have signed on informed consent.

### Sampling procedures

The Needle of 22 gauges was used for sampling. The aspiration samples were obtained from 1–2 passes in different directions of nodes, thereby the yield of tumor cells was further increased. The needle was removed from the lesion, and a small amount of sample was placed on a glass slide to make a conventional smear and staining for diagnosis. The remaining specimen was rinsed into a vial of cytolyt (Hologic Corp.) and made into a ThinPrep slide (Hologic Corp., Thinprep 5000). The slides were stained with the papanicolaou staining method for diagnosis and estimating the amount of tumor cells. The suspension sample was ready for molecular test. If the tumor cells were not enough for molecular test (estimated by the following tumor cells quantity estimation), for the palpable nodes, we performed FNA again until an adequate number of neoplastic cells were acquired. Re-FNA was required in one-third cases approximately. For EBUS-FNA samples, we did not require re-FNA because of inconvenient clinical operation. Therefore, only the adequate samples were selected in this study.

In 19 cases, FFPE (Formalin-Fixed and Parrffin-Embedded) samples were obtained for detection as contrast. the FFPE tissue and cytologic samples were also obtained from the same lymph node in 10 cases. The cytologic samples were from the lymph node and the tissue samples from primary site of lung for the remaining 9 cases.

### The estimation of tumor cells quantity

The cytologic slides were reviewed by cytopathologists to determine the quality and quantity of tumor cells for molecular testing. First, we selected the cases diagnosed as adenocarcinoma by FNA cytology, some diagnosis were made in combination with immunocytochemistry. The morphological equivocal cases without immunocytochemistry results were excluded from our study. Second, sufficient tumor cells were confirmed. We assessed tumor cell quantity according to the smears and the volume of suspensions in the preservative solution. The specimens met the following criteria in this study: 1) the estimated ratio is based on the tumor cell amount. For each case, the tumor cell percent was at least 20% and at least 300 tumor cells were present in the slide [9]. 2) Those cases must contain no less than 10 ml of residual preservative solution (Hologic Corp.) and white granular suspensions can be observed by naked eye in the phial as well.

### DNA extraction

The residual LBC material in the preservative solution was submitted for molecular testing within 1–3 days in normal temperature. After centrifuging, the deposits were used for DNA extraction. DNA was extracted using QIAamp^®^ DNA Mini Kit (Qiagen Corp., Germany), according to the manufacturer’s instructions. Genomic DNA was extracted from 19 cases of paraffin-embedded tumor tissues using QIAamp^®^ DNA Mini Kit (Qiagen, Germany), according to the manufacturer’s instructions. The quality and concentration of the DNA samples were examined by NanoDrop (Thermo Corp.).

### Real-time PCR

The real-time PCR was performed using the *EGFR* and *KRAS* Mutation Test Kit (ACCB, Beijing, China). In some cases, the EGFR mutation detection was performed by real time PCR and NGS simultaneously, which showed consistent result (See Supplementary Table 1). This data indicated that our real-time PCR technique is reliable. DNA was adjusted to a fixed concentration and added to the detection mixture. The target DNA was then amplified and detected on the Stratagene Mx3000P analyzer (Agilent Technologies Inc, Santa Clara, CA). The following PCR procedure was used: an initial denaturation at 95°C for 10 min and 40 cycles of 95°C for 15 s, 60°C for 1 min to perform the data collection. The result judgment was according to the fusion fluorescence signal.

### Statistical analysis

Patient characteristics were examined with descriptive statistics. The age was analyzed using independent samples *t*-test. χ^2^ test was used to compare the mutations rate. Consistency test was used for paired comparition. *P* value < 0.05 was considered significant.

### Ethics approval

This study were reviewed and approved by the ethics committee of the Cancer Hospital, CAMS. All patients had written informed consent.

## SUPPLEMENTARY MATERIALS TABLE



## Abbreviations

FNA: fine-needle aspiration
NSCLC: Non-small cell lung cancer
*EGFR*: epithelial growth factor receptor
TKIs: tyrosine kinase inhibitors
LBC: liquid-based cytology
real-time PCR: real-time polymerase chain reaction
EBUS-TBNA: endobronchial ultrasound guided transbronchial needle aspiration
CR: complete response
PR: partial response
SD: stable disease
PD: progress disease
ORR: Objective response rate
FFPE: Formalin-Fixed and Parrffin-Embedded
