# Supplementary material for: Detection of EGFR and KRAS gene mutations using suspension liquid-based cytology specimens in metastatic lung adenocarcinoma
Source: Oncotarget. 2017 Nov 20;8(63):106685–92. doi: 10.18632/oncotarget.22530 (PMC5739766; doi:10.18632/oncotarget.22530)
Supplement: Supplementary file 1 [file oncotarget-08-106685-s001.pdf]

Detection of *EGFR* and *KRAS* gene mutations using suspension liquid-based cytology specimens in metastatic lung adenocarcinoma

SUPPLEMENTARY MATERIALS

Supplementary Table 1: List of cases for *EGFR* mutation detection between real time PCR and NGS

Sample ID	Real time PCR	NGS
2	Exon 20 c.2369C>T(p.T790M)	Exon 20 c.2369C>T(p.T790M)
12	Negative	Negative
16	Negative	Negative
22	Negative	Negative
62	Negative	Negative
92	Exon 19 deletion	Exon 19 c.2236_2250del15 (p.E746_ A750delELREA)
115	Negative	Negative
124	Exon 21 c.2573T>G(p.L858R)	Exon 21 c.2573T>G(p.L858R)
132	Negative	Negative
139	Exon 19 deletion and exon 20 c.2369C>T(p.T790M)	Exon 19 c.2236_2250del15 (p.E746_ A750delELREA) and exon 20 c.2369C>T(p.T790M)
